# Increased Postoperative Fasting Glucose Is Associated With Unfavorable Outcomes in Patients Treated With Mechanical Thrombectomy Treatment

**DOI:** 10.3389/fneur.2021.668363

**Published:** 2021-05-28

**Authors:** Zongjie Shi, Shunyuan Guo, Jie Pan, Chao Xu, Yu Geng, Sujie Zheng

**Affiliations:** ^1^Zhejiang Provincial People's Hospital, Hangzhou, China; ^2^Hangzhou Medical College, Hangzhou, China

**Keywords:** stroke, fasting glucose, mechanical thrombectomy, outcome, stent

## Abstract

**Background and objective:** Hyperglycemia on admission was associated with worse clinical outcomes after mechanical thrombectomy (MT) of acute ischemic stroke (AIS). We evaluated whether increased postoperative fasting glucose (PFG) was also related to poor clinical outcomes in patients who underwent MT treatment.

**Methods:** Consecutive patients with large vessel occlusion underwent MT in our center were included. Admission glucose and fasting glucose levels after MT treatment were evaluated. Primary outcome was 90-day unfavorable outcomes (modified Rankin Scale score of 3–6). Secondary outcome was the rate of symptomatic intracranial hemorrhage (sICH) after MT treatment. The association of PFG and 90-day clinical outcome after MT treatment was determined using logistic regression analyses.

**Results:** One hundred twenty seven patients were collected. The median postoperative fasting glucose level was 6.27 mmol/L (IQR 5.59–7.62). Fourteen patients (11.02%) had sICH, and fifty-eight patients (45.67%) had unfavorable outcomes at 90-day after MT. After adjustment for potential confounding factors, PFG level was an independent predictor of 90-day unfavorable outcome (OR 1.265; 95% CI 1.017–1.575; *p* = 0.035) and sICH (OR 1.523; 95% CI 1.056–2.195; *p* = 0.024) after MT. In addition, older age, higher baseline NIHSS score, and higher postoperative NLR were also associated with unfavorable outcomes at 90-day after MT treatment.

**Conclusions:** Increased PFG is associated with unfavorable outcomes at 90-day and an increased risk of sICH in patients underwent MT treatment.

## Introduction

Endovascular mechanical thrombectomy (MT) therapy is now accepted as standard treatment for stroke patients caused by large vessel occlusions in anterior circulation (1). However, about 54% of patients still have a poor prognosis, even after urgent and successful reperfusion (2).

Hyperglycemia is common and independently associated with worse clinical outcomes in AIS patients (3). The potential effects of hyperglycemia include increased lactic acidosis resulting in increasing cytotoxic edema and impairment of the penumbra, reduced cerebral vasomotor reactivity and damage collateral circulation, disruption of the blood-brain barrier and increased risk of sICH (4–8). Several studies found that hyperglycemia on admission or fasting hyperglycemia were associated with worse outcomes in patients treated with intravenous alteplase (9, 10). However, whether disturbed glucose metabolism is related to clinical outcome in patients after MT is unknown. Recently, several studies have focused on the influence of admission glucose on outcomes for endovascular therapy. Huo et al. showed that higher admission blood glucose was independently associated with poor clinical outcome at 3 months in patients experienced MT (11). Goyal et al. showed that higher admission glucose and hyperglycemia were independent predictors of worse functional outcomes in patients treated with MT (12).

Previous research has mainly focused on the effects of admission glucose or hyperglycemia and clinical outcomes after MT, but the correlation between PFG and prognosis remains unclear. In this study, we explored the potential association between PFG level and 90-day clinical outcomes in patients treated with MT.

## Methods

### Study Population

We retrospectively collected a consecutive group of patients who treated with MT at Zhejiang Provincial People's Hospital between August 2015 and April 2017. The inclusion criteria were (1) Patients with AIS and admission within 6 h after symptom onset; (2) Proximal large vessel occlusion in the intracranial internal carotid artery, the middle cerebral artery M1 or M2; and (3) treatment with MT and successful reperfusion. Patients were excluded if they (1) had posterior circulation large vessel occlusion or (2) lacked integral laboratory data, such as glucose levels at different time points or subsequent CT/MRI scans.

This study was authorized by the Ethics Committee of Zhejiang Provincial People's Hospital (2017KY021).

### Data Collection and Clinical Assessment

Demographics, medical history, clinical and laboratory data, and procedural characteristics were collected for analysis. Stroke severity was measured by National Institutes of Health Stroke Scale (NIHSS). Stroke etiology was classified by the criteria of the Trial of ORG 10172 in Acute Stroke Treatment (13). Successful reperfusion was defifined as a modifified Thrombolysis in Cerebral Infarction (mTICI) score of 2b or 3 (14). Symptomatic intracranial hemorrhage (sICH) was diagnosed according to Heidelberg Bleeding Classification criteria (15): (1) neurological deterioration ≥4 total points on NIHSS; (2) NIHSS increase of ≥2 points in any subcategory; (3) led to intubation, hemicraniectomy, external ventricular drain placement; (4) absence of other explanation for deterioration. The mRS at 90-day was obtained by outpatient or telephone follow-up. Clinical outcomes at 90-day after MT was divided into a favorable outcome (mRS 0–2) and an unfavorable outcome (mRS 3–6).

### Statistical Analysis

Kolmogorov-Smirnov test was performed to examine the normality of distribution. Continuous variables were presented as the mean ± standard deviation (SD, normal distribution) and the median with IQR (skewed distribution). Differences between patients with and without favorable outcomes were explored using Student's *t* test or Mann-Whitney *U* test for continuous variables and Fisher's exact test or χ2 test for categorical variables. Clinically variables with *p* < 0.05 from the results of the univariate analyses were included in logistic regression. Adjustments were performed for age, baseline NIHSS, post-operation NIR (neutrophil-lymphocyte ratio, NLR), a multivariate logistic regression was used to analyze the postoperative fasting glucose on 90-day clinical outcomes. After adjusting for baseline ASPECTS, platelet count, IV-tpa, hypertension, admission glucose, a multivariate logistic regression was used to analyze the postoperative fasting glucose on sICH after MT. A two-tailed *P* < 0.05 was deemed statistically signifificant. Statistical analysis was conducted using IBM SPSS (version 25).

## Results

Overall, 127 patients were included in the current analysis. The mean age was 70.95 years, and 51.2% were men. The median baseline NIHSS score was 20 (IQR 16–25), and the median time from stroke onset to treatment was 305 min (IQR 236–366). Median PFG levels were 6.27 mmol/L (IQR 5.59–7.62). Fourteen patients (11.02%) had sICH. A total of fifty-eight patients (45.67%) had unfavorable outcomes at 90 days. The main characteristics of the patients are summarized in Table 1.

**Table 1:** Comparison of characteristics between patients with favorable and unfavorable outcome.

	**All patients**	**Favorable outcome**	**Unfavorable outcome (*n* = 58)**	***P***
	**(*n* = 127)**	**(*n* = 69)**		
Age (y), mean ± SD	70.95 ± 12.24	67.38 ± 13.04	75.21 ± 9.73	0.001
Male, *n* (%)	65 (51.2)	40 (58)	25 (43.1)	0.095
Baseline NIHSS, median (IQR)	20 (16–25)	18 (16–24)	24 (20–28)	0.001
**Preexisting conditions**, ***n*** **(%)**				
Smoking	19 (15)	10 (52.6)	9 (15.5)	0.872
Hypertension	86 (67.7)	44 (63.8)	42 (72.4)	0.299
Diabetes mellitus	16 (12.6)	7 (10.1)	9 (15.5)	0.363
Atrial fibrillation	90 (70.9)	44 (63.8)	46 (79.3)	0.055
**Vital signs (mm Hg), median (IQR)**				
Baseline SBP	146 (132–160)	146 (131.5–158)	147.5 (135.5–165.5)	0.262
Baseline DBP	85 (75–96)	85 (74–95.5)	84.5 (76–96)	0.521
Post-operation SBP	127 (112–140)	128 (114.5–140)	125 (110–144.5)	0.545
Post-operation DBP	75 (65–83)	76 (64–86)	74.5 (65–81.25)	0.764
**Laboratory finding, median (IQR)**				
Platelet count(*10^9^/L)	159 (126–191)	163 (128–191)	155 (124.75–191.5)	0.474
Admission glucose (mmol/L)	6.77 (6.21–8.32)	6.85 (6.11–8.07)	6.77 (6.29–8.71)	0.289
Postoperative fasting glucose (mmol/L)	6.27 (5.59–7.62)	6.12 (5.28–6.95)	7.03 (6.02–9.17)	0.001
Baseline NLR	5.87 (3.43–9.48)	6.29 (3.58–9.06)	5.35 (3.33–10.04)	0.742
Post-operation NLR	7.89 (5.77–12.38)	7.2 (4.95–9.29)	9.33 (6.51–15.75)	0.001
Uric acid (μmol/L)	281 (236–340)	287 (239.5–356.5)	276 (231.7–337.75)	0.487
Creatinine (μmol/L)	65 (55.4–77)	63 (52.75–75.75)	68.39 (57.4–77.73)	0.392
**Procedure time(min), median (IQR)**				
Onset to door	210 (141–274)	211 (109.5–298.5)	207 (143.25–265.5)	0.713
Onset to puncture	305 (236–366)	321 (229–376.5)	289 (236.75–343.5)	0.239
Door to puncture	99 (64–122)	102 (80.5–124.5)	89 (58.75–114)	0.057
Onset to reperfusion	360 (295–418)	383 (286.5–431)	349 (312.75–404.75)	0.335
IV tPA administration, *n* (%)	53 (41.7)	30 (43.5)	23 (39.7)	0.663
General anesthesia, *n* (%)	76 (59.8)	37 (53.6)	39 (67.2)	0.183
Baseline ASPECTS, median (IQR)	8 (6–10)	8 (7–10)	7 (5–8)	0.001
**Location of occlusion site**, ***n*** **(%)**				0.091
ICA	42 (33.1)	19 (27.5)	23 (39.7)	
M1-MCA	74 (58.3)	41 (59.4)	33 (56.9)	
M2-MCA	11 (8.7)	9 (13)	2 (3.4)	
**TOAST classification**, ***n*** **(%)**				0.092
Large arterial atherosclerosis	15 (11.8)	12 (17.4)	3 (5.2)	
Cardioembolism	98 (77.2)	49 (71)	49 (84.5)	
Undetermined etiology	14 (11)	8 (11.6)	6 (10.3)	
**SICH**, ***n*** **(%)**	14 (11)	2 (2.9)	12 (20.7)	0.001

*Mean ± SD, mean (standard deviation, SD); n (%), number(percent); IQR, interquartile range; NIHSS, National Institutes of Health Stroke Scale; SBP, systolic blood pressure; DBP, diastolic blood pressure; NLR, neutrophil-lymphocyte ratio; IV tPA, intravenous tissue-type plasminogen activator; ASPECTS, indicates Alberta Stroke Program Early CT Score; ICA, internal carotid artery; M1-MCA, M1 segment of middle cerebral artery; M2-MCA, M2 segment of middle cerebral artery; TOAST, the trial of Org 10172 in acute stroke treatment; SICH, symptomatic intracerebral hemorrhage*.

Compared to patients without favorable outcomes, patients with favorable outcomes tended to be younger (67.38 vs. 75.21 years old, *p* = 0.001). Moreover, patients with favorable outcomes had lower baseline NIHSS scores (median, 18 vs. 24; *P* = 0.001), lower postoperative fasting glucose levels (median, 6.12 vs. 7.03; *P* = 0.001), lower postoperative NLR (7.2 vs. 9.33; *p* = 0.001), and higher baseline ASPECTS (median, 8 vs. 7; *P* = 0.001). The prevalence of sICH was lower in patients with favorable outcomes (2.9% vs. 20.7%; *P* = 0.001) (Table 1).

After adjusting for confounding factors (age, baseline NIHSS score, postoperative NLR) in multivariate logistic analyses, PFG level remained an independent predictor of 90-day unfavorable outcome after MT (OR 1.265; 95% CI 1.017–1.57); *p* =0.035). We also found that older age, higher baseline NIHSS score, and higher postoperative NLR were also associated with unfavorable outcomes at 90-day after MT (Table 2).

**Table 2:** Association between postoperative fasting glucose and 90-day unfavorable outcomes after mechanical thrombectomy.

	**OR (95%CI)**			
	**Unadjusted**	***p***	**Adjusted**	***p***
Age	1.062 (1.026–1.100)	0.001	1.053 (1.011–1.096)	0.013
Baseline NIHSS	1.136 (1.063–1.215)	0.001	1.112 (1.035–1.195)	0.004
Post-operation NLR	1.156 (1.068–1.251)	0.001	1.128 (1.038–1.225)	0.004
Post-operative fasting glucose	1.421 (1.159–1.741)	0.001	1.265 (1.017–1.575)	0.035

*NIHSS, National Institutes of Health Stroke Scale; NLR, neutrophil-lymphocyte ratio*.

After adjusting for aseline ASPECTS, platelet count, IV-tpa, hypertension, admission glucose, multivariate regression analyses showed that PFG (OR 1.523; 95% CI 1.056–2.195; *p* = 0.024) was an independent risk factor for sICH after MT (Table 3).

**Table 3:** Association between postoperative fasting glucose and sICH after mechanical thrombectomy.

	**OR (95%CI)**			
	**Unadjusted**	***p***	**Adjusted**	***p***
Baseline ASPECTS	0.657 (0.481–0.897)	0.008	0.712 (0.472–1.074)	0.105
Platelet count	0.981 (0.968–0.995)	0.010	0.978 (0.959–0.998)	0.035
IV tPA	4.070 (1.201–13.787)	0.024	6.253 (1.309–29.870)	0.022
Hypertension	0.219 (0.068–0.705)	0.011	0.198 (0.041–0.951)	0.043
Admission glucose	1.308 (1.013–1.689)	0.040	1.127 (0.790–1.609)	0.509
Postoperative fasting glucose	1.563 (1.229–1.988)	0.000	1.523 (1.056–2.195)	0.024

*ASPECTS, indicates Alberta Stroke Program Early CT Score; IV tPA, intravenous tissue-type plasminogen activator*.

## Discussion

In this study, we found that increased postoperative fasting glucose level was independently associated with unfavorable outcomes at 90-day and an increased risk of sICH in patients treated with mechanical thrombectomy. We speculated that hyperglycemia should be managed more aggressively after MT.

Previous research has demonstrated that hyperglycemia was independently associated with adverse clinical outcomes in AIS, and a J-shaped association between admission glucose and clinical outcomes has been reported in patients with AIS before the endovascular treatment era (16). Compared to previous therapy, MT has significantly improved the recanalization rate and clinical prognosis, and the impact of hyperglycemia on clinical outcomes in patients underwent MT requires further research. Observational study shown that hyperglycemia maybe an adverse prognostic factor in patients treated with MT (17). Borggrefe et al. showed that admission hyperglycemia, age, and NIHSS score were main factors for an unfavorable outcome after MT (18). Rinkel et al. showed that increased admission glucose was associated with worse outcome and increased risk of sICH (19). Broocks G and colleagues found that elevated admission blood glucose levels were associated with aggravation brain edema and adverse clinical outcomes (20). The impact of blood glucose levels on outcome depended on collateral status, and higher glucose levels reduced the likelihood of a favorable outcome among patients with good collaterals but less significantly for patients with poor collaterals (21). However, the influence of admission hyperglycemia on clinical outcomes have shown contradictory results. In MR CLEAN pretrial cohort, there were no association between admission blood glucose level and 90-day functional outcome was found (22).

To date, the association of admission hyperglycemia and poor outcome in patients with AIS after MT has been well-studied. However, only a few studies have evaluated the role of impaired fasting glucose on outcomes in patients treated with MT. Our study findings are consistent with recently published studies that found that increasing PFG levels were independently associated with unfavorable outcomes at 90-day and an increased risk of sICH after MT. Osei et al. reported that fasting glucose after MT was an independent risk factor for worse outcomes in patients experienced MT (23). Li et al. reported that postoperative glucose levels might be an independent risk factor for sICH in patients with acute large vessel occlusion who are treated with MT (24). Increased fasting glucose levels are a result of stress hyperglycemia, which is caused by the interplay of hormones, with concomitant insulin resistance during acute illness (25). Therefore, fasting glucose, which reflects stress hyperglycemia, may be more reliable in predicting clinical outcomes. One study found that higher fasting glucose levels after intravenous alteplase predicted poor outcome or death better than admission glucose; moreover, the association of fasting glucose with unfavorable outcome was independent of HBA1c levels and the presence of diabetes (26). Another study supported than fasting glucose was a more important predictor of long-term clinical outcome in patients treated with MT compared with glucose on admission (27).

The impact of dysfunctional glucose metabolism on clinical outcomes after MT could be explained by several pathophysiological mechanisms. Hyperglycemia may affect mitochondrial function in ischemic penumbra. Altered mitochondrial function leads to acidosis and cell death (5); impairs cerebrovascular reactivity in the microvasculature, which may disturb reperfusion after recanalization (28); and alters blood barrier permeability and induces blood barrier disruption, which may aggravate brain edema formation and lead to hemorrhagic transformation (4).

In addition, consistent with prior research, we confirmed that a higher postoperative NLR was also associated with unfavorable outcomes at 90-day after MT. Moustafa Aly et al. showed that a lower NLR at 3–7 days was an independent predictor of favorable outcomes and reduced risk for sICH (29). Semerano et al. found that the follow-up higher NLR in MT patients was associated with an increased risk of sICH (30). The underlying mechanism may involve the activation of neutrophils and the suppression of lymphocytes by systemic stress, resulting in an increased risk of reperfusion injury, malignant edema, and/or hemorrhagic transformation (31, 32).

Our study has several limitations. First, the study design was retrospective with all inherent limitations, and we adjusted the variables using multivariate logistic regression analysis to exclude possible bias. Second, this study only includes data from this period, as the cohort is relatively small, a longer time frame and thus more patients would increase the power of the statistical findings. Third, the effect of blood glucose level on outcome depended on collateral status, and collateral grades were not evaluated in our data. Fourth, glucose levels are a dynamic condition, and one isolated value at one point seems to be insufficient.

## Conclusion

In conclusion, our study revealed that increased postoperative fasting glucose seems to be associated with unfavorable outcomes at 90-day and an increased risk of sICH in AIS patients treated with MT. These findings indicate the importance of optimal management of serum glucose after MT in AIS patients with large vessel occlusion.

## Data Availability Statement

The original contributions presented in the study are included in the article/supplementary material, further inquiries can be directed to the corresponding authors.

## Ethics Statement

The studies involving human participants were reviewed and approved by this study was approved by the Ethics Committee of Zhejiang Provincial People's Hospital (Approval No: 2017KY021). The patients/participants provided their written informed consent to participate in this study.

## Author Contributions

ZS: substantial contributions to study design, data collection for the whole trial, data analysis and interpretation of data, drafting and revising the manuscript for intellectual content, and wrote the statistical analysis plan. CX: substantial contributions to study design, cleaned and analyzed the data, revising the manuscript for intellectual content, image data collection for the whole trial, and revising the manuscript for intellectual content. SG and JP: data acquisition, interpretation of data, and revising the manuscript for intellectual content. SZ and YG: study concept and design, study supervision, interpretation of data, revising the manuscript critically for intellectual content, and final approval of the version to be published. All authors agree to be accountable for all aspects of the work in ensuring that questions related to the accuracy or integrity of any part of the work are appropriately investigated and resolved.

## Conflict of Interest

The authors declare that the research was conducted in the absence of any commercial or financial relationships that could be construed as a potential conflict of interest.
